# A proteolysis-targeting chimera molecule selectively degrades ENL and inhibits malignant gene expression and tumor growth

**DOI:** 10.1186/s13045-022-01258-8

**Published:** 2022-04-08

**Authors:** Xin Li, Yuan Yao, Fangrui Wu, Yongcheng Song

**Affiliations:** 1 Department of Pharmacology and Chemical Biology, Baylor College of Medicine, 1 Baylor Plaza, Houston, TX 77030 USA; 2 Dan L. Duncan Comprehensive Cancer Center, Baylor College of Medicine, 1 Baylor Plaza, Houston, TX 77030 USA

**Keywords:** PROTAC, ENL, MLL1-rearranged leukemia, ENL mutation, Cancer therapeutics

## Abstract

**Background:**

Chromosome translocations involving mixed lineage leukemia 1 (MLL1) cause acute leukemia in most infants and 5–10% children/adults with dismal clinical outcomes. Most frequent MLL1-fusion partners AF4/AFF4, AF9/ENL and ELL, together with CDK9/cyclin-T1, constitute super elongation complexes (SEC), which promote aberrant gene transcription, oncogenesis and maintenance of MLL1-rearranged (MLL1-r) leukemia. Notably, ENL, but not its paralog AF9, is essential for MLL1-r leukemia (and several other cancers) and therefore a drug target. Moreover, recurrent ENL mutations are found in Wilms tumor, the most common pediatric kidney cancer, and play critical roles in oncogenesis.

**Methods:**

Proteolysis-Targeting Chimera (PROTAC) molecules were designed and synthesized to degrade ENL. Biological activities of these compounds were characterized in cell and mouse models of MLL1-r leukemia and other cancers.

**Results:**

Compound **1** efficiently degraded ENL with DC_50_ of 37 nM and almost depleted it at ~ 500 nM in blood and solid tumor cells. AF9 (as well as other proteins in SEC) was not significantly decreased. Compound **1**-mediated ENL reduction significantly suppressed malignant gene signatures, selectively inhibited cell proliferation of MLL1-r leukemia and Myc-driven cancer cells with EC50s as low as 320 nM, and induced cell differentiation and apoptosis. It exhibited significant antitumor activity in a mouse model of MLL1-r leukemia. Compound **1** can also degrade a mutant ENL in Wilms tumor and suppress its mediated gene transcription.

**Conclusion:**

Compound **1** is a novel chemical probe for cellular and in vivo studies of ENL (including its oncogenic mutants) and a lead compound for further anticancer drug development.

**Supplementary Information:**

The online version contains supplementary material available at 10.1186/s13045-022-01258-8.

## Introduction

Acute lymphocytic leukemia (ALL) and myeloid leukemia (AML) caused by chromosome translocations of mixed lineage leukemia 1 (MLL1, also known as MLL or KMT2A) account for ~ 70% of the diseases in infants and 5–10% in children and adults with a poor prognosis [1–3]. Five-year survival rates for MLL1-rearranged (MLL1-r) ALL are ~ 35% [4–7], as compared with ~ 90% for other pediatric ALLs. MLL1-r AML also carries poor clinical outcomes with five-year survivals of ~ 30% [8, 9]. Although a significant progress has been achieved to understand the biology of MLL1-r leukemias [1, 10], more effective treatments are needed.

Despite > 70 fusion partners of MLL1 were identified, only a few are frequently found in ~ 70% MLL1-r leukemias [1, 10–12], including transcription cofactors AF9 (also known as MLLT3) [13] and its paralog ENL (also known as MLLT1) [14], AF4 and its paralog AFF4 [15, 16], and ELL (Additional file 1: Figure S1a). Together with the cyclin-T1/CDK9 complex (also known as P-TEFb), these proteins associate with each other and constitute super elongation complexes (SEC) [15–17], which promote malignant gene expression (e.g., HoxA9, Meis1 and Myc) in MLL1-r leukemia and play critical roles in the cancer initiation and maintenance (Additional file 1: Figure S1b).

**Fig. 1:**
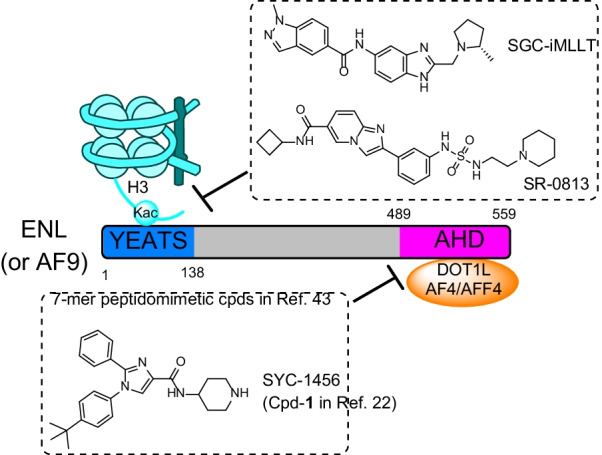
Illustration of ENL and its paralog AF9 with associated proteins. The representative inhibitors of these protein–protein interactions are also shown

ENL and homologous AF9 contain an N-terminal YEATS, a central intrinsically disordered linker and C-terminal AHD domain (Fig. 1). The YEATS domain recognizes an acetylated histone lysine residue (e.g., H3K27ac) and such binding has been found to be important in gene regulation [18, 19]. The less conserved, long linker regions of ENL and AF9 have been poorly studied. While YEATS is lost in most clinical variances of MLL1-AF9/-ENL and dispensable for the leukemia, the AHD domain is always present in the fusion oncogenes and required for leukemogenesis [15]. Recognizing a consensus sequence of LxVxIxLxxV/L, ENL/AF9 AHD can bind AF4/AFF4 or histone H3K79 methyltransferase DOT1L [20, 21] with a high affinity (Additional file 1: Figure S1b) [22–24]. Thus, in addition to forming SEC for transcription elongation, AHD can recruit DOT1L for hypermethylation of H3K79, which is characteristic and critical to MLL1-r leukemia [25–30]. Moreover, ENL/AF9 AHD can also bind CBX8 (chromobox homolog 8) [31, 32] or BCoR (BCL-6 corepressor) [33] and such protein–protein interactions have been reported to be important for MLL1-AF9/-ENL mediated leukemogenesis [31, 32, 34, 35]. Interestingly, despite their high homology (particularly in the YEATS and AHD domains), ENL functions differently from AF9 with knockout studies showing that ENL, but not AF9, is critical to MLL1-r leukemia and other AMLs [19, 36].

Recently, recurrent mutations in the YEATS domain of ENL have been found in Wilms tumor, the most common pediatric kidney cancer [37]. Dysregulated expression of certain Hox genes and Myc is characteristic of ENL-mutated Wilms tumors. Further studies show the mutation-induced self-association of the mutant ENL and there was significantly increased binding of the mutant ENL-associated SEC to these gene loci, causing aberrant gene transcription and eventually oncogenesis [38].

Much interest has been generated to pharmacologically inhibit ENL/AF9. Several potent small-molecule inhibitors of YEATS were reported to disrupt the ENL/AF9-H3K27ac interaction [39–42]. Several 7-mer peptidomimetic compounds [43] and a small-molecule compound SYC-1456 [22] are inhibitors of the AHD domain. SYC-1456 (developed by us) can suppress onco-MLL1 mediated aberrant gene expression, induce cell differentiation and apoptosis, and inhibit tumor growth in cell and mouse models of MLL1-r leukemia, thereby validating ENL inhibition is a viable therapeutic approach [22].

Proteolysis-targeting chimera (PROTAC) technology has recently attracted much interest in drug discovery [44]. Featuring good cell permeability, a PROTAC molecule may cause proteasome-mediated degradation of its target protein, which complements pharmacological inhibition with a distinct mechanism of action. It also has other potential benefits, such as sub-stoichiometric activity and more selectivity [44]. Here, we report a PROTAC molecule that can cause efficient and selective degradation of ENL (but not AF9), resulting in inhibition of malignant gene signatures and proliferation of MLL1-r leukemia in vitro and in vivo.

## Results

### Compound design and synthesis

**Fig. 2:**
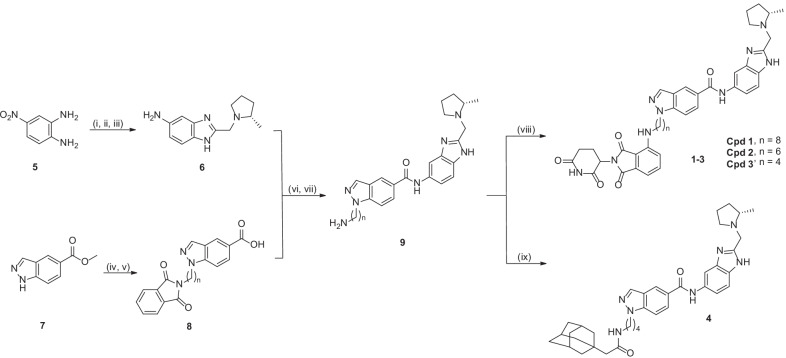
Structures and synthesis of compounds **1**–**4** with reagents and conditions: (i) Ethyl chloroacetate, 4 N HCl (aq.), 100 °C, 94%; (ii) (*2S*)-2-Methylpyrrolidine, Na_2_CO_3_, CH_3_CN, 70%; (iii) H_2_, 10% Pd/C, MeOH; (iv) NaH, *N*-(6-bromohexyl)phthalimide, dimethylformamide, 47.7%; (v) 37% HCl (aq.), 100 °C; (vi) 1-[Bis(dimethylamino)methylene]-1H-1,2,3-triazolo[4,5-b]pyridinium 3-oxide hexafluorophosphate, diisopropylethylamine, dichloromethane, 25 °C, 65%; (vii) NH_2_NH_2_, EtOH, 50 °C, 89%; (viii) 2-(2,6-dioxo-3-piperidinyl)-4-fluoro-1*H*-isoindole-1,3(2*H*)-dione, diisopropylethylamine, dimethyl sulfoxide, 90 °C, 44%; (ix) 1-adamantaneacetic acid, condition vi, 64.4%

Our designed PROTAC molecules consist of a YEATS inhibitor SGC-iMLLT [39] and covalently linked thalidomide, a commonly used ligand of E3 ubiquitin ligase Cereblon (Additional file 1: Figure S2) [44]. It is expected that upon binding to ENL, the PROTAC compound can recruit Cereblon through its thalidomide moiety to form a ternary complex for ubiquitination of ENL, which is subjected to proteasome-mediated degradation. Based on the X-ray structure of ENL in complex with SGC-iMLLT [39], we designed compounds **1**–**3** (Fig. 2) with their linkers having no steric conflicts with ENL. In addition, a hydrophobic tagging [45] compound **4** was designed as the second strategy to degrade ENL.

Synthesis of these compounds is shown in Fig. 2. 4-Nitrobenzene-1,2-diamine (**5**) was reacted with ethyl 2-chloroacetate to give 2-chloromethyl-5-nitrobenzimidazole, which was subjected to a substitution reaction with (*S*)-2-methylpyrrolidine followed by reduction of -NO_2_ to give compound **6**. Substitution reaction between methyl 1*H*-indazole-5-carbonate (**7**) and *N*-(6-bromohexyl)phthalimide followed by hydrolysis produced compound **8**, which was coupled with **6** to give, upon deprotection, compound **9**. A nucleophilic substitution reaction between the -NH_2_ of **9** and the *ortho*-F-substituted thalidomide afforded the target PROTAC compounds **1**–**3**. An amide-forming reaction between **9** and 1-adamantaneacetic acid produced compound **4**.

### ENL-targeting PROTACs bind to ENL/AF9 YEATS

Using an ALPHA (amplified luminescent proximity homogeneous assay) assay [39], compounds **1**–**3** were evaluated for their inhibition of the ENL YEATS-H3K27ac interaction. Compound **1** strongly inhibited such protein–protein interaction with an IC_50_ of 170 nM (Additional file 1: Table S1), while it is weaker than the parent inhibitor SGC-iMLLT (IC_50_ = 32 nM in our assay). Similarly, compounds **2** and **3** are also strong inhibitors (IC_50_ = 100 and 610 nM). These results indicate the linker-thalidomide moieties of compounds **1**–**3** only slightly reduce the binding affinity of the SGC-iMMT to ENL YEATS. The decreased affinity might be due to the entropy costs associated with the flexibly linked thalidomide. In addition, consistent with previous studies [39], compounds **1**–**3** were found to bind to AF9 YEATS with comparable affinities (Additional file 1: Table S1) using a similar ALPHA assay.

### Cpd-1 efficiently degrades ENL, but not its paralog AF9

**Fig. 3:**
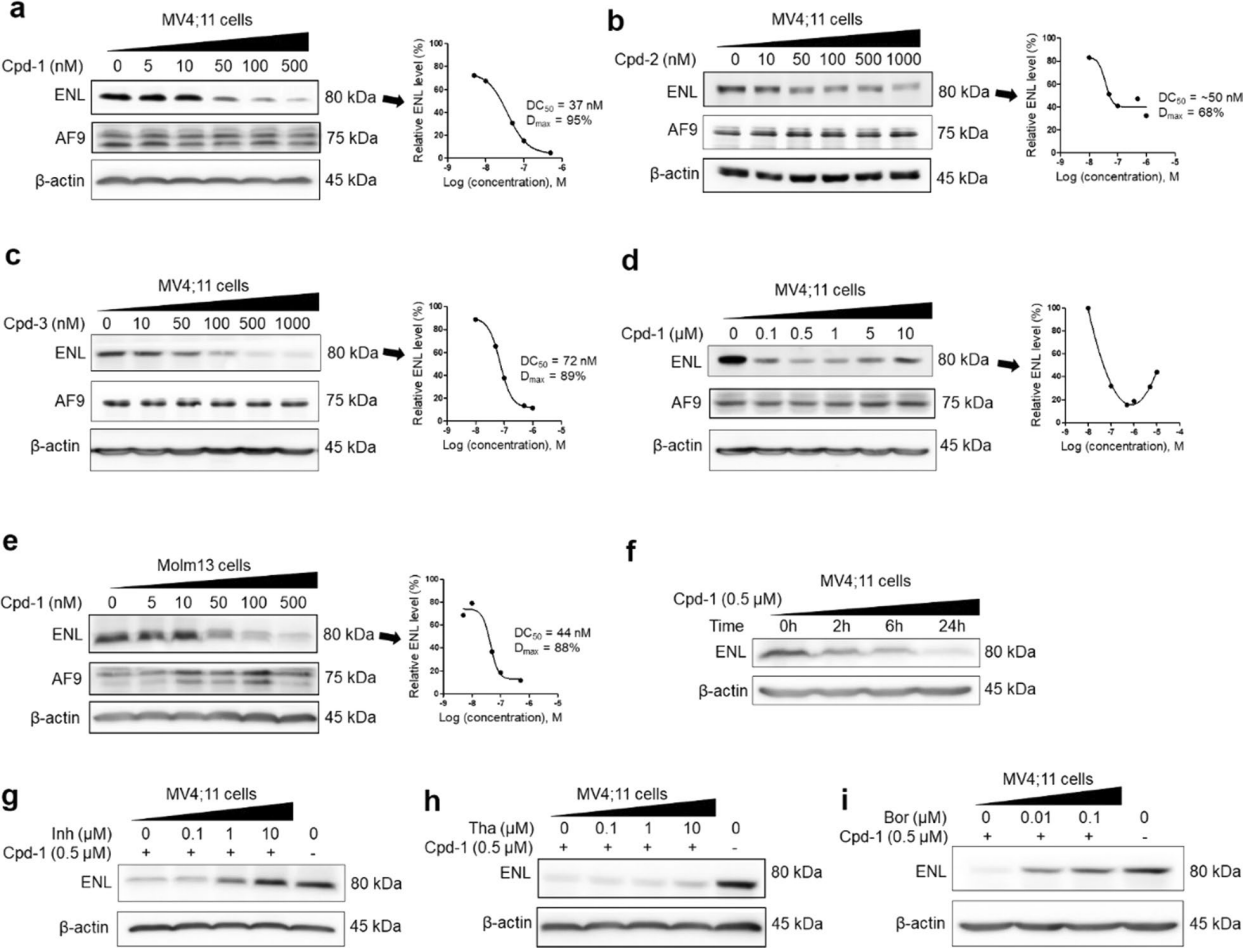
Activity of compounds **1**–**3** on ENL and AF9 in MLL1-r leukemia cells. **a**–**e** Levels of ENL, AF9 and β-actin (as a control) in **a**–**d** MV4;11 and **e** Molm-13 cells upon treatment with compounds **1** (**a**/**d**/**e**), **2** (**b**), and **3** (**c**) at the specified concentrations for 24 h, showing they induced degradation of ENL with their dose-responsive curves for calculating DC_50_ shown at right. AF9 levels were not reduced; **f** time-dependent degradation of ENL in MV4;11 cells by **1** (500 nM); **g**–**i** ENL levels in MV4;11 cells upon pre-treatment for 2 h with SGC-iMLLT (Inh, **g**), thalidomide (Tha, **h**) and bortezomib (Bor, **i**) followed by co-treatment with **1** (500 nM) for 24 h, showing these three compounds can dose-dependently inhibit **1**-mediated ENL degradation

We next tested whether these compounds degrade ENL and AF9 in MV4;11 leukemia cells with the MLL1-AF4 oncogene. Upon compound treatment for 24 h, the cells were washed, lysed, and the lysates subjected to SDS-PAGE (sodium dodecyl sulfate–polyacrylamide gel electrophoresis) followed by detection with Western blot. As shown in Fig. 3a, c, compounds **1** and **3** efficiently degraded ENL in a dose-dependent manner with DC_50_ (concentration at which a protein is reduced by 50%) values of 37 and 72 nM, respectively, and almost depleted it at ~ 500 nM with D_max_ (maximal degradation) of ~ 95% and 89%. While compound **2** can degrade ENL with a DC_50_ of ~ 50 nM, it only reduced ENL by a maximum of ~ 68% (Fig. 3b). Compound **4** did not reduce ENL even at 5 μM (Additional file 1: Figure S3). Notably, AF9 levels in all these experiments were not reduced, showing compounds **1**–**3** are selective ENL-degrading probes.

The most active compound **1** was chosen for additional studies. It was found to exert the maximal activity at ~ 500 nM, but the ENL level started to recover at higher concentrations from 1–10 μM (Fig. 3d). This “hook” effect is commonly observed for a PROTAC [44, 46], because excessive compound **1** elevates inactive binary complexes ENL-**1** and Cereblon-**1**, and decreases the active ternary complex ENL-**1**-Cereblon. In addition, compound **1** efficiently degraded ENL in another MLL1-r leukemia Molm-13 cells (with MLL1-AF9) with DC_50_ of 47 nM and D_max_ of ~ 88% (Fig. 3e). Moreover, significant ENL degradation can be detected in 2 h and depletion occurred within 24 h (Fig. 3f).

Mechanistically, SGC-iMLLT or thalidomide competitively inhibits the binding of **1** to ENL or Cereblon, respectively, while proteasome inhibitor bortezomib suppresses proteasome’s activity to degrade ENL. All of these compounds should impair compound **1**’s ability to degrade ENL. MV4;11 cells were pre-treated with these three compounds for 2 h, followed by co-treatment with compound **1** (500 nM) for 24 h. As shown in Fig. 3g–i, compound **1**’s ability to degrade ENL was compromised by the three compounds in a dose-dependent manner. These rescue experiments support compound **1** is a PROTAC-based ENL degrader.

### Cpd-1 only reduces ENL, but not other SEC proteins in cells or gene promoters

**Fig. 4:**
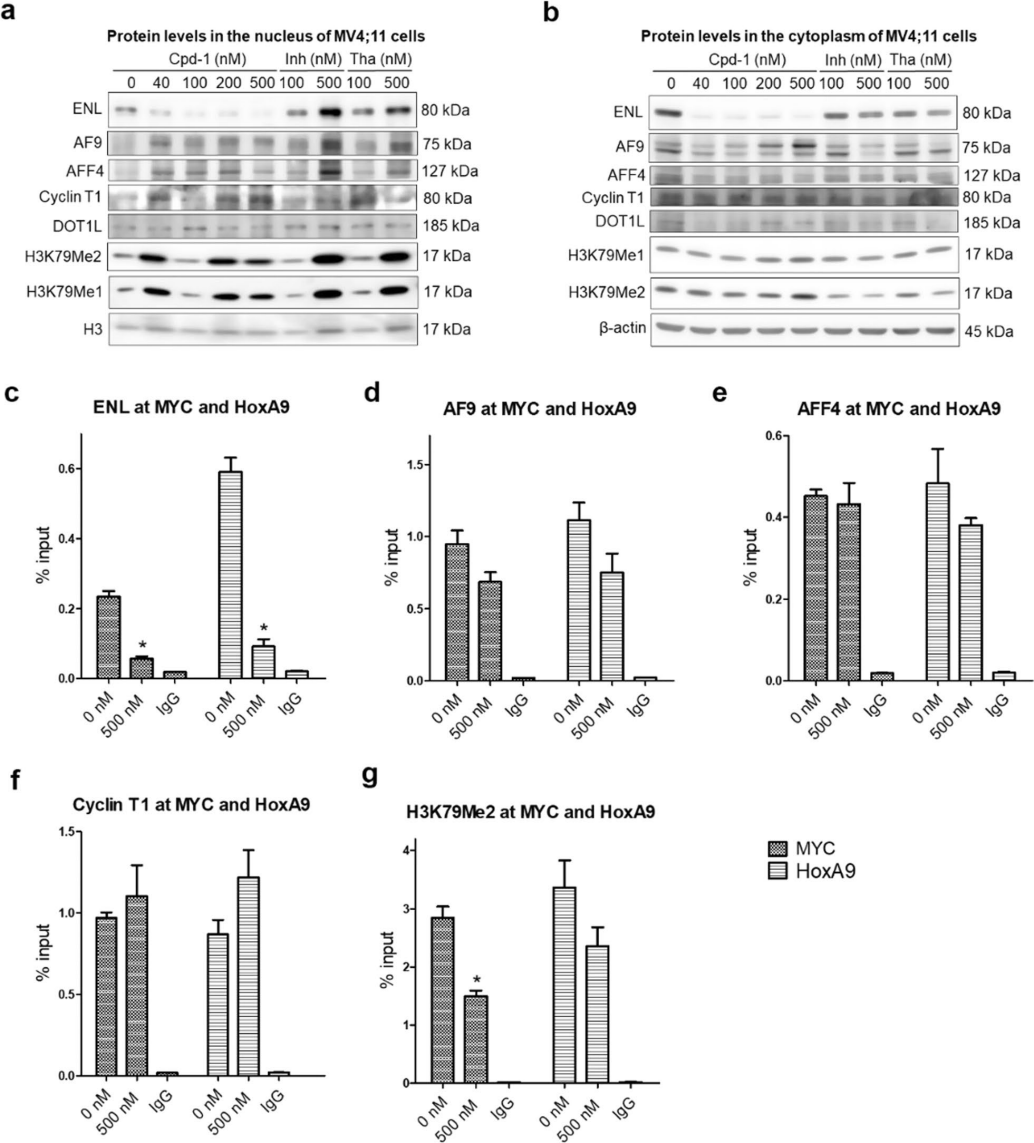
Compound **1** reduced ENL, but not other SEC proteins in MLL1-r leukemia cells. **a**, **b** Upon compound treatment at the specified concentrations for 4 days, levels of ENL and other related proteins in the **a** nucleus and **b** cytoplasm of MV4;11 cells, showing **1** only significantly reduced ENL; **c**–**g** ChIP-qPCR results showing the enrichment of **c** ENL, **d** AF9, **e** AFF4, **f** cyclin-T1 and **g** H3K79me2 in the gene promoters of Myc and HoxA9. Treatment with compound **1** (500 nM) only significantly reduced the binding of ENL to these gene promoters. (*p < 0.05)

We further investigated how compound **1** affects ENL, AF9 and other proteins of SEC in the cytoplasmic and nuclear compartments, the latter of which are more relevant to the functions of these transcription cofactors. SGC-iMLLT and thalidomide were included as controls for possible off-target effects. Upon treatment of MV4;11 cells with these compounds for 4 days, cytoplasmic and nuclear proteins were separated and subjected to SDS-PAGE/Western blot. As shown in Fig. 4a, ENL in the nucleus was significantly reduced dose-dependently and almost depleted with 500 nM of compound **1**. SGC-iMLLT caused no reduction or even an increase in nuclear ENL, presumably because the inhibitor binds and helps stabilize ENL. Thalidomide did not reduce nuclear ENL. Moreover, AF9, AFF4 and cyclin-T1, three major components of SEC, as well as DOT1L (which binds AF9/ENL), were not significantly decreased by compound **1**. In addition, nuclear levels of H3K79 methylation, the product of DOT1L catalyzed reactions, were not consistently affected by compound **1**. The observed H3K79 methylation variations are puzzling but seem to be caused by off-target effects, as SGC-iMLLT or thalidomide caused similar changes. Similar protein changes were observed for the cytoplasmic extract, with only ENL levels were significantly reduced by compound **1** (Fig. 4b).

Chromatin immunoprecipitation (ChIP) followed by qPCR was used to further probe the activity of compound **1** in the gene promoters of Myc and HoxA9, two characteristic MLL1 target genes. As shown in Fig. 4c–g, compound **1** significantly reduced the ENL levels at these gene promoters, but in general, it did not significantly lower down AF9, AFF4, cyclin-T1 and H3K79me2 at these gene loci.

### Cpd-1-mediated ENL degradation suppresses malignant gene signatures in MLL1-r leukemia

**Fig. 5:**
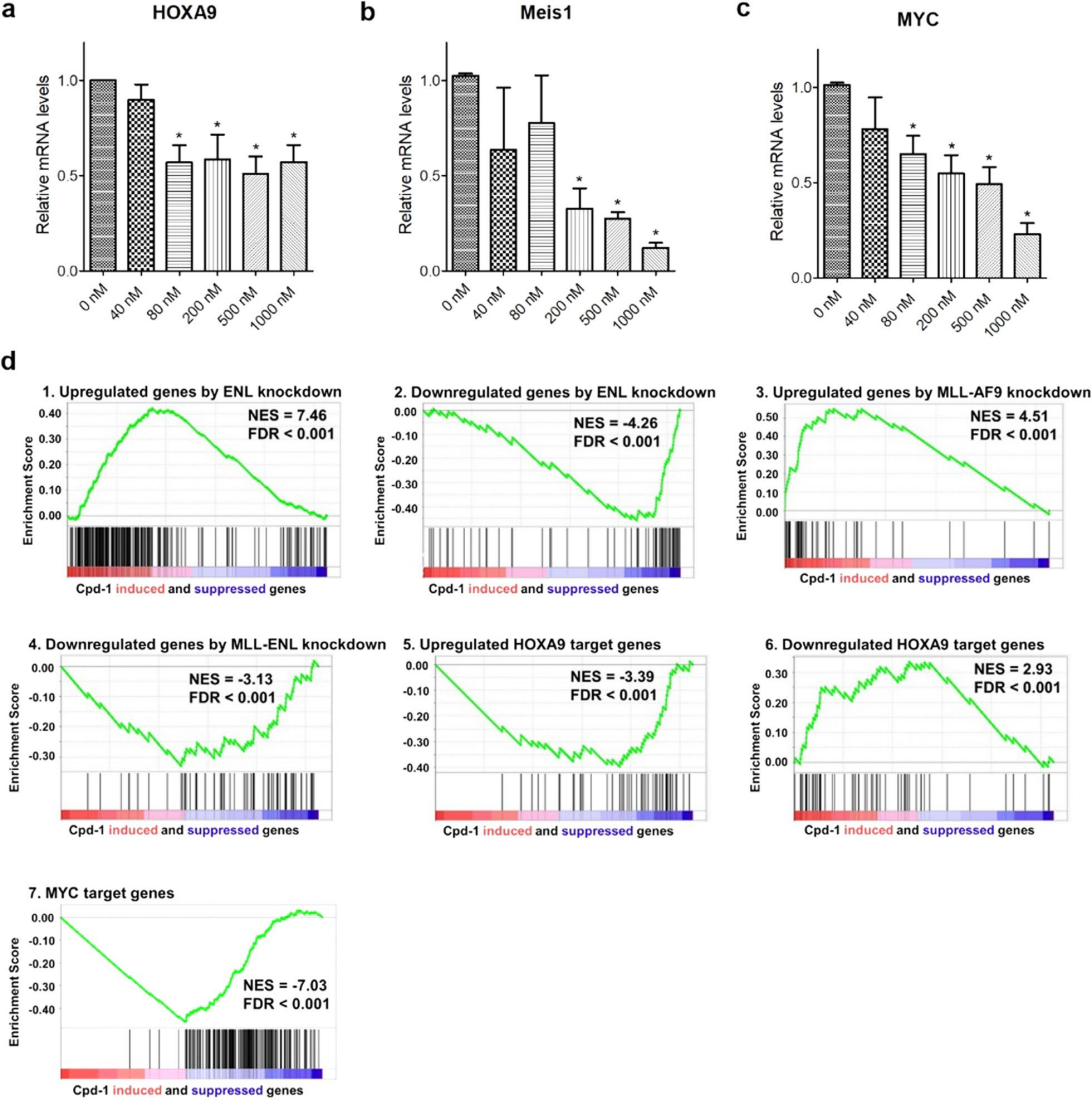
Compound **1** inhibited malignant gene expression in MLL1-r leukemia Molm-13 cells. **a**–**c** Treatment with **1** for 4 days dose-dependently inhibited expression of **a** HoxA9, **b** Meis1, and **c** Myc (*p < 0.05); **d** Gene profiling followed by gene set enrichment analysis (GSEA) shows that treatment of Molm-13 cells with compound **1** (500 nM for 4 days) recapitulated activities of (1, 2) ENL knockdown (GSE80774) and (3, 4) knockdown of MLL1-AF9 and MLL1-ENL (GSE36592 and Ref. 48). It also significantly (5, 6) reversed expression of HoxA9-regulated target genes (GSE13714), and (7) downregulated Myc target genes (GSE32220)

Previous studies show ENL is required for expression of MLL1-target genes in MLL1-r leukemia [15, 19]. How compound **1**-mediated ENL degradation changes expression of HoxA9, Meis1 and Myc, three characteristic genes in MLL1-r leukemia [22], was examined. Molm-13 cells were treated with compound **1** for 4 days, after which the RNAs were extracted and analyzed. As shown in Fig. 5a–c, compound **1** was able to significantly inhibit expression of these three genes in a generally dose-dependent manner.

Gene profiling was performed to find how compound **1** affects global gene transcriptome. mRNAs from the control and compound **1** (500 nM) treated Molm-13 cells were extracted, purified and sequenced. Bioinformatic analysis was performed to find differentially expressed genes between the treated and control cells, which were used for gene set enrichment analysis (GSEA). The results are shown in Fig. 5d. Compound **1** caused significant upregulation of a gene set that was upregulated upon knockout of ENL [19], with normalized enrichment score (NES) of 7.46 and false discovery rate (FDR) of < 0.001 (Fig. 5d.1). It also led to significant downregulation of a gene set that was downregulated upon ENL knockout [19] with NES of − 4.26 and FDR of < 0.001 (Fig. 5d.2). These clearly indicate that treatment with compound **1** recapitulated ENL knockout with similar patterns of gene expression changes. In addition, compound **1** significantly upregulated and downregulated the gene sets that were upregulated and downregulated by knockdown of MLL-AF9 [47] or -ENL [48] (Fig. 5d.3 and 4), showing the compound treatment mimicked knockdown of these fusion oncogenes. Moreover, compound **1** counteracted two critical transcription factors HoxA9 and Myc in MLL1-r leukemia: treatment with compound **1** reversed expression patterns of HoxA9-regulated genes [49] (Fig. 5d.5 and 6). It also significantly downregulated transcription of Myc target genes [50] (Fig. 5d.7). These results are consistent with ENL’s critical roles in MLL1-r leukemia and show compound **1** acted on-target.

### Cpd-1 inhibits cell proliferation, induces differentiation and apoptosis of MLL1-r leukemia cells

**Fig. 6:**
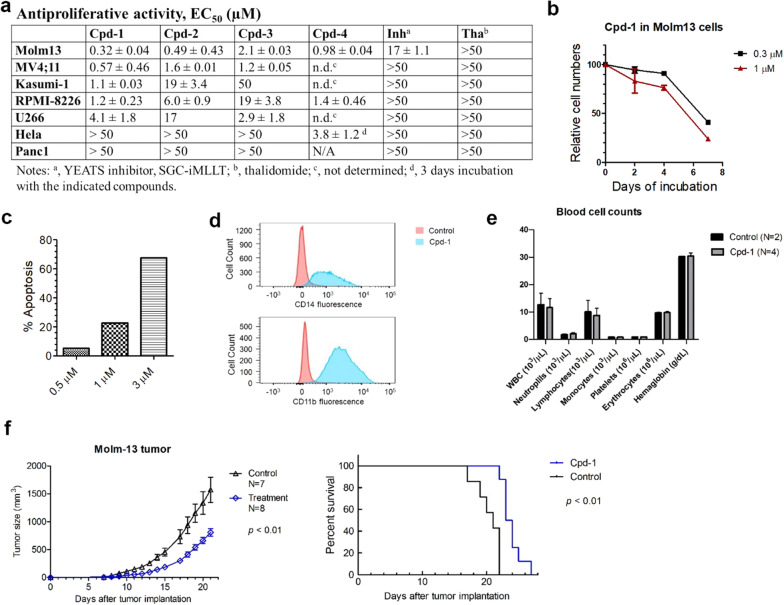
Antitumor activities of ENL-targeting compounds. **a** Antiproliferative activities of compounds upon 7-day incubation, showing compound **1** inhibited proliferation of MLL1-r leukemia (Molm-13 and MV4;11) and Myc-driven Kasumi-1 (AML) and RPMI8226 and U266 (myeloma) cells, while it had no activity against solid Hela (cervical) and Panc1 (pancreatic) cancer cells; **b** Time-dependent activity of compound **1** against proliferation of Molm-13 cells; **c**, **d** Treatment of Molm-13 cells with compound **1** led to **c** dose-dependent apoptosis and **d** differentiation (at 3 μM) with more cells expressing high levels of CD14 (upper) and CD11b (lower); **e** Treatment with compound **1** (30 mg/kg/day for 13 days) caused no significant changes in blood cell counts; **f** Treatment with **1** (30 mg/kg/day for Day-3–15) significantly inhibited tumor growth (left) with prolonged survivals (right) in mice with subcutaneously xenografted Molm-13 leukemia

Compound **1** exhibited potent activity against proliferation of MLL1-r leukemia cells Molm-13 and MV4;11 with EC_50_ values of 320 and 570 nM (Fig. 6a and Additional file 1: Figure S4), while the parent inhibitor SGC-iMLLT and thalidomide were inactive (EC_50_ > 50 μM) except that SGC-iMLLT had weak activity against Molm-13 cells. Compound **1** also showed strong activity (EC_50_ = 1.1–4.1 µM) against AML Kasumi-1 and myeloma RPMI-8226 and U266 cells, in which Myc is critical. However, solid tumor cells Hela (cervical) and Panc1 (pancreatic) are insensitive to compound **1** with EC_50_ of > 50 µM. The selective antitumor activities of **1** are consistent with the critical functions of ENL (or SEC) in MLL1-r leukemia and Myc-driven cancers [51]. Less active compounds **2** and **3** possess a similar antitumor profile with generally reduced potencies (Fig. 6a). Compound **4**, which failed to degrade ENL, exhibited strong, but non-selective activities (EC_50_ = 1.0–3.8 µM) for these blood and solid tumor cells, presumably due to off-target effects.

As with many compounds targeting gene expression (e.g., epigenetic inhibitors of DOT1L or LSD1 [26, 28, 52]), compound **1** exhibited a slow action against cell proliferation. It did not inhibit cell proliferation during the first 4 days, but showed potent activity upon a longer treatment (Fig. 6b and Additional file 1: Fig. S5). In contrast, compound **4** is a cytotoxic agent, killing cancerous cells non-selectively within 3 days. These results support that the antiproliferative activity of compound **1** is on-target: it degrades ENL and causes suppression of aberrant gene expression and eventually inhibition of cell proliferation at a later stage.

Treatment of Molm-13 cells with compound **1** for 7 days at 1 and 3 µM caused significant apoptosis of 22.7% and 67.6%, respectively (Fig. 6c and Additional file 1: Fig. S6). No significant apoptosis (≤ 5%) was observed at 0.5 μM or with a shorter incubation (e.g., for 4 days). Compound **1** (3 μM for 5 days) also induced cell differentiation, with significantly more cells having high levels of CD14 and CD11b, two cell surface proteins characteristic to differentiated macrophages or monocytes (Fig. 6d).

### Cpd-1 inhibits tumor growth in a mouse model of MLL1-r leukemia

In vivo antitumor activity of compound **1** was evaluated in a commonly used mouse model of Molm-13 leukemia [53, 54]. First, in vivo toxicity was assessed in C57BL/6 mice. Treatment with compound **1** (30 mg/kg/day for 13 days) did not cause significant weight losses (Additional file 1: Figure S7) as well as any visible signs of toxicity. A blood test on day-14 showed that there were no significant differences in blood cell counts between mice in the treatment and control groups (Fig. 6e). These results suggest compound **1** at this dosage did not inhibit normal hematopoiesis or cause other overt toxicities to mice. Next, 10^6^ Molm-13 cells were injected subcutaneously into NOD-SCID mice, which developed palpable tumors in ~ 1 week and grew rapidly. As shown in Fig. 6f, treatment with compound **1** (30 mg/kg/day for Day 3–15) significantly inhibited tumor growth in mice with prolonged animal survivals (*p* < 0.01). Similarly, it did not cause significant weight losses in these animals (Additional file 1: Figure S8).

### Cpd-1 also degraded mutant ENL and suppressed its mediated gene transcription

**Fig. 7:**
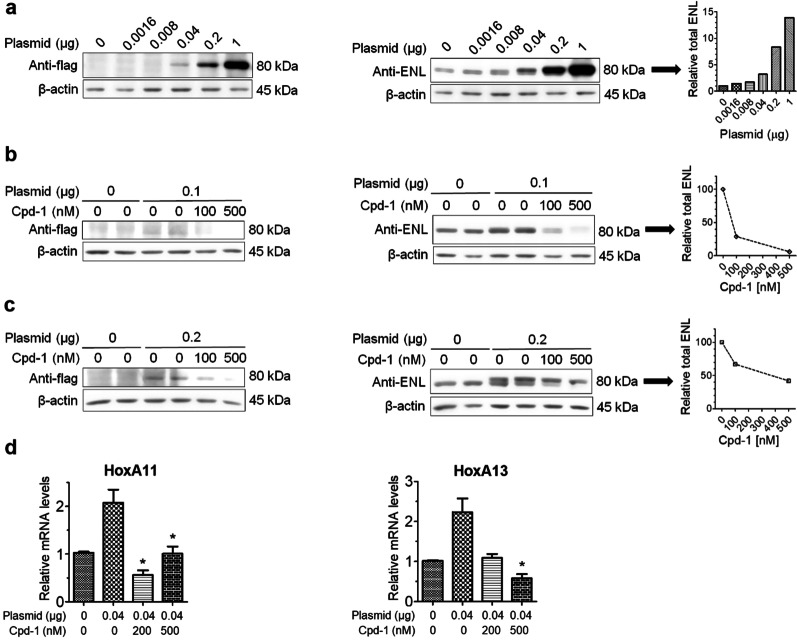
Compound **1** degraded mutant ENL (mENL) and suppressed its mediated gene transcription. **a** Cellular levels of mutant/wild-type (WT) ENL and β-actin detected by (left panel) FLAG or (right panel) ENL antibody, upon transfection with increasing amounts of a mENL-containing plasmid for 4 h followed by 24 h incubation, showing dose-dependent expression of mENL. Endogenous WT ENL can also be detected and included in quantification (right panel); **b**, **c** Levels of mutant/WT ENL detected by a FLAG or ENL antibody, upon transfection with **b** 0.1 or **c** 0.2 μg of the plasmid for 4 h followed by 24 h treatment with compound **1**, showing the compound can dose-dependently degrade or deplete both mutant and WT ENL; **d** Transfection with 0.04 μg of the plasmid for 4 h followed by 24 h incubation upregulated expression of HoxA11 (left) and HoxA13 (right), and treatment with **1** during the incubation inhibited such gene overexpression (*p < 0.05)

Ability of compound **1** to degrade mutant ENL, which has been implicated to cause Wilms tumor, was evaluated. Frequent clinical ENL mutants contain a short in-frame insertion or deletion in the YEATS domain [38], but they retain similar binding affinities to the parent inhibitor SGC-iMLLT [55]. A pcDNA3.1(+)-N-DYK plasmid containing a mutant ENL (mENL) with a short insertion of -NHL- between L117 and R118 [38] was transfected into 5 × 10^5^ HEK293T cells. Upon incubation for 24 h, expression of the FLAG-tagged mENL can be dose-dependently detected with as low as 0.04 μg of the plasmid (Fig. 7a). Using a FLAG antibody appeared to be less quantitative because of a higher background staining for the control samples (Fig. 7a–c, left panels). While both endogenous, wild-type (WT) ENL and mENL (which cannot be separated by SDS-PAGE) can be detected by an ENL antibody recognizing the peptide residues surrounding A343, the blots had a clean background for quantification (right panels).

With 0.1 μg of the plasmid, compound **1** can efficiently degrade both WT and mutant ENL proteins with *D*_max_ of ~ 95% at ~ 500 nM upon 24 h incubation (Fig. 7b). With 0.2 μg of the plasmid, compound **1** (500 nM) can still degrade WT and mutant ENLs, but with a reduced efficiency (Fig. 7c). It was ineffective with 1 μg of the plasmid (not shown), presumably because the rate of mENL synthesis in cells was higher than that of **1**-mediated degradation. Since mENL expression in Wilms tumor is comparable to that of WT ENL in normal tissues [38] and 0.2 μg of the plasmid produced considerably more mENL than endogenous ENL (Fig. 7a, right), it is expected that compound **1** can efficiently degrade and even deplete mENL in Wilms tumor.

Expression of mENL has been found to upregulate certain Hox genes, such as HoxA11 and HoxA13, in Wilms tumor and drive oncogenesis [38]. Next, we investigated how mENL degradation affect expression of HoxA11 and HoxA13 in this cell model. Upon transfection with 0.04 μg of the plasmid followed by 24 h incubation, expression of HoxA11 and HoxA13 was found to be significantly upregulated (Fig. 7d). Treatment with compound **1** during the incubation significantly inhibited overexpression of HoxA11 and HoxA13 (Fig. 7d), showing degradation of mENL could downregulate aberrant gene expression in Wilms tumor.

## Discussion

MLL1-r leukemia found in the majority of infant and 5–10% of children/adult patients is a distinct subtype of acute leukemia with poor clinical outcomes. The current treatment options are chemotherapeutics, which non-selectively kill all rapidly proliferating cells including normal stem/progenitor cells and cause toxicities, side effects, and even therapy-related secondary cancers. Although several novel compounds, such as inhibitors of DOT1L, LSD1 and BRD4, are in clinical trials [3], there have been no effective treatments for MLL1-r leukemia. Less toxic, targeted therapies are therefore needed.

Together with the cyclin-T1-CDK9 complex (P-TEFb), the most frequent MLL1 fusion partners AF4/AFF4, ENL/AF9 and ELL associate with each other on the AF4/AFF4 heterodimeric scaffold [10] and constitute SEC, which play essential roles in malignant gene expression, oncogenesis and maintenance of MLL1-r leukemia [15–17]. Knockout studies show ENL is required for MLL1-r leukemia and several other AMLs [19, 36], but its paralog AF9 bearing highly homologous YEATS and AHD domains is dispensable, indicating ENL has additional critical functions in these cancers. However, ENL and AF9 seem to be equally important for SEC-mediated HIV gene expression [56]. In addition, ENL knockout had no or minimal effects on normal hematopoietic stem cells as well as solid tumor cells [19, 36]. These lines of evidence support selective inhibition of ENL represents a promising and potentially less toxic therapy for MLL1-r leukemia and possibly other cancers.

Several potent small-molecule inhibitors of the YEATS domain of ENL/AF9 (Fig. 1) have been reported with low-nM biochemical activity [39–42], but none of these compounds showed strong antitumor activities in cells or animal models. SYC-1456, our recently disclosed inhibitor of the AHD domain of ENL/AF9 [22], exhibited strong antitumor activities (with low-μM EC_50_s) in MLL1-r leukemia cells and mouse models. An SR-0813 (Fig. 1)-derived PROTAC molecule had relatively weak effects in degrading ENL/AF9 without antitumor activity due to its poor stability [40]. Notably, none of these chemical probes exhibit high selectivity between ENL and AF9.

In this study, compound **1** was found to be a highly efficient, ENL-specific PROTAC molecule, able to degrade ENL with DC_50_ as low as 37 nM and deplete it at ~ 500 nM (*D*_max_ ~ 95%) in a variety of blood and solid tissue cells (Figs. 3 and 7). AF9 (as well as other proteins in SEC) was not significantly reduced (Fig. 4a, b). ChIP experiments further indicate it only reduced the ENL levels in several MLL1 target gene promoters (Fig. 4c). Compound **1**-mediated ENL depletion significantly suppressed aberrant gene signatures in MLL1-r leukemia, including reduced expression of several characteristic genes (e.g., HoxA9 and Myc) (Fig. 5). **1**-mediated global gene expression changes caused inhibited cell proliferation (with EC_50_s as low as 320 nM) and cell differentiation and apoptosis. It also showed significant antitumor activity in a mouse model of MLL1-r leukemia (Fig. 6f). These results are consistent with ENL’s essential roles in MLL1-r leukemia and other cancers (e.g., Myc-driven cancers) and support AF9 is indeed dispensable in these contexts. Thus, compound **1** is the first potent chemical probe for cellular and in vivo studies of ENL’s functions in health and diseases. It also represents a pharmacological lead for future drug development for these cancers.

The high selectivity of compound **1** might stem from different number or proximity of the surface-accessible lysine residues between ENL and AF9 [57]. Sequence alignment of ENL and AF9 (Additional file 1: Figure S9) indicates ENL possesses 6 more lysine residues in a short peptide segment 175–192 in its intrinsically disordered region, while few different lysine residues are in the conserved YEATS and AHD domains. This ENL-specific, Lys-enriched segment might be preferentially ubiquitinated by **1**-bound Cereblon, causing selective ENL degradation.

Moreover, compound **1** was found to degrade mENL with similar activity. Because other clinical ENL mutants in Wilms tumor exhibited comparable binding affinities to SGC-iMLLT [55] and such mutations are far away from the Lys-enriched region, compound **1**’s ability to degrade other mutant ENLs is expected. Therefore, compound **1** is a useful chemical probe for ENL-mutated Wilms tumor.

## Conclusion

We developed a potent PROTAC molecule **1** for selective ENL degradation. It strongly inhibited malignant gene expression and cell proliferation of MLL1-r leukemia and Myc-driven cancers. Compound **1** is a novel probe for cellular and in vivo studies of ENL (including cancer-associated ENL mutants) and a lead compound for further anticancer drug development.

## Methods

### Compound synthesis and characterization

All chemicals for synthesis were purchased from Alfa Aesar (Ward Hill, MA) or Aldrich (Milwaukee, WI). The identity of the synthesized compounds was characterized by ^1^H and ^13^C NMR on a Varian (Palo Alto, CA) 400-MR spectrometer and mass spectrometer (Shimadzu LCMS-2020). The identity the most potent compounds was confirmed with high resolution mass spectra (HRMS) using an Agilent 6550 iFunnel quadrupole-time-of-flight (Q-TOF) mass spectrometer with electrospray ionization (ESI). The purities of the final compounds were determined to be > 95% with a Shimadzu Prominence HPLC using a Zorbax C18 (or C8) column (4.6 × 250 mm) monitored by UV at 254 nm. Synthesis and characterization of compounds can be found in Supplemental Material.

### Plasmids and peptides

cDNA for human ENL YEATS domain (1–138) was synthesized (by Genscript) and cloned into pET-28a vector. The H3K27Ac peptide [Biotin-AHX-RKQLATKAARK(Ac)S] was purchased from Genscript. cDNA for mENL was synthesized (by Genscript) and cloned into pcDNA3.1(+)-N-DYK vector.

### Protein expression and purification

The expression plasmids were used to transform *E. coli* BL21(DE3) strain (Novagen, USA), and protein expression was induced in the presence of 0.4 mM isopropyl β-d-1-thiogalactopyranoside (IPTG) at 16 °C overnight. Cells were collected and lysed using French pressure cell press (GlenMills, USA) in lysis buffer: 50 mM NaH_2_PO_4_, 300 mM NaCl, 20 mM imidazole, pH 7.8. Upon centrifugation, the supernatant was applied to a HisTrap (GE Healthcare, USA) nickel column and the protein was eluted with a linear imidazole gradient from 20 to 250 mM. The resultant protein solution was then subjected into a size exclusion column (HiLoad 16/60 Superdex 75, GE Healthcare) to get the purified protein (> 95%, SDS-PAGE).

### Alpha assay

AlphaScreen binding assay was developed using Perkin-Elmer AlphaScreen Histidine (Nickel Chelate) Detection Kit, following a reported protocol [39]. Data were imported into Prism (version 5.0), and IC50 values from 3 independent experiments with standard deviation were obtained by using a standard dose–response curve fitting.

### Western blot

3 × 10^6^ cells/well were treated with increasing concentrations of a compound for 1 day, and whole proteins were extracted. Equal amounts of proteins were separated on SDS-PAGE and transferred to PVDF membranes. The blots were probed with primary antibodies, followed by anti-rabbit IgG (Thermo Scientific) secondary antibodies. The primary antibodies against ENL (Cell signaling #14893), AF9 (Novusbio #NB100-1565), DOT1L (Cell signaling #77087), AFF4 (Abcam #ab103586), Cyclin T1 (Cell signaling #81464), H3K79me2 (Cell signaling #5427), H3K79me1 (Cell signaling #9398), Histone H3 (Cell signaling #4499), FLAG (Sigma-Aldrich #F1804), β-Actin (Cell signaling #4970) were used in this study.

### Antiproliferation assay

Proliferation inhibition assays for suspension blood cancer cells were performed using an XTT assay kit (Biotium), following our previous methods [22]. EC50 values were determined using Prism 5 and from at least three independent experiments.

### Flow cytometry

For Annexin V apoptosis assay, 10^5^ cells/mL were incubated with increasing concentrations of a compound for 7 days. Apoptosis was determined using the FITC Annexin V Apoptosis Detection Kit I (BD Bioscience) using the manufacturer’s protocol. For other FACS assays, cells were labeled with fluorochrome-conjugated monoclonal antibodies against human CD14 and CD11b (BD Biosciences) according to the manufacturer’s recommendation. Cells were analyzed using a FACS Calibur (BD Biosciences/Applied Biosystems), and data were processed using the program Flowjo (version7.6.5).

### RNA extraction and quantitative real-time PCR (qPCR)

10^5^ cells/mL were incubated with a compound for 4 days and the RNA was extracted using RNeasy mini kit (#74104, Qiagen). 100–1000 ng of total RNA was reverse transcribed using iScript™ Reverse Transcription Supermix (Bio-Rad) using the manufacturer’s protocol. Quantitative real-time PCR was carried out using Fast SYBR Green Master Mix (Applied Biosystems) according to the manufacturer’s instructions. Measurements were performed in triplicate, using GAPDH as the reference gene. Real-time PCR was performed using Biosystems Step One Plus detection system. The following sequences of primers are used:

- MYC (forward: 5′-CACCGAGTCGTAGTCGAGGT-3′; reverse: 5′-TTTCGGGTAGTGGAAAACCA-3′);
- HoxA9 (forward: 5′-TACGTGGACTCGTTCCTGCT-3′; reverse: 5′-CGTCGCCTTGGACTGGAAG-3′);
- Meis1 (forward: 5′-CCAGCATCTAACACACCCTTAC-3′; reverse: 5′-TATGTTGCTGACCGTCCATTAC-3′);
- GAPDH (forward: 5′-GCGAGATCCCTCCAAAATCAA-3′; reverse: 5′-GTTCACACCCATGACGAACAT-3′).

### Chromatin immunoprecipitation

Upon treatment with a compound for 4 days, 10^7^ MV4;11 cells were cross-linked with 1% formaldehyde at room temperature for 10 min, followed by the addition of 125 mM glycine. Cells were lysed with nuclear lysis buffer and sonicated to ~ 100–1000 bp fragments, which was incubated at 4 °C overnight with an antibody and IgG (C15410206, Diagenode). Protein A/G Magnetic Beads (10 µL, Novus Biologicals) were added and incubated for 2 h. The beads were washed 3 × with RIPA buffer and 2 × with TE buffer. DNA on the beads was eluted for 2 h at 68 °C in 100 μL of an elution buffer (20 mM Tris pH 7.5, 5 mM EDTA, 50 mM NaCl, 1% SDS, 50 μg/mL proteinase K) (2 ×), and purified using a ChIP DNA Clean & Concentrator kit (Novus Biologicals). qPCR was done using the method described above.

### Library preparation, clustering and sequencing

Library preparation for RNA-sequencing was performed using our previous reported methods [22]. Cluster generation of the denatured libraries was performed utilizing the HiSeq X PE Cluster Kit V2.5 (Illumina) according to the manufacturer’s instructions. Sequencing was performed on a Novaseq6000 sequencer (Illumina) using S4 flowcell with paired-end 101 bp reads and a 6 bp index read culminating in an average output of 45 million paired-end reads per sample. Sequence read data were processed and converted to FASTQ format by Illumina BaseSpace analysis software (v2.0.13).

### Bioinformatics analysis

The pair-ended reads were mapped to the human genome (UCSC hg19) using software STAR (https://github.com/alexdobin/STAR) with NCBI RefSeq genes as the reference. The gene-based read counts generated by STAR were used as the measurement for gene expression. R Bioconductor package DESeq2 (http://bioconductor.org/) was used to analyze the gene-based read counts to detect differentially expressed genes between the groups of interest. The false discovery rate (FDR) of the differentially expressed genes was estimated using Benjamini and Hochberg method. FDR < 0.05 was considered statistically significant. Gene set enrichment analysis (GSEA) was performed using the GSEA software (https://www.gsea-msigdb.org/gsea/index.jsp).

### In vivo* antitumor studies*

All of the mouse studies were conducted in strict compliance with an IRB-approved protocol. NOD-SCID mice (6–8 weeks old, from Jackson lab) were obtained and maintained under specific pathogen-free conditions. 10^6^ Molm13 cells in medical grade saline were inoculated subcutaneously, and palpable tumors (2–3 mm in diameter) were developed in ~ 1 week. Mice were treated with compound **1** (30 mg/kg/day for 13 days) in saline (0.1 mL) administered intraperitoneally. Tumors were measured every day and estimated by using the formula a × b^2^/2.

### Transfection with mENL

5 × 10^5^ HEK293T cells were transfected with mENL containing pcDNA3.1(+)-N-DYK plasmid using jetPRIME (Polyplus Transfection) following the manufacturer's protocol. Upon transfection for 4 h, the media were carefully removed and the cells incubated with fresh media containing the specified concentrations of compound **1** for 24 h before further analysis (using Western blot or qPCR as described above).

### Statistical analysis

At least three independent experiments were carried out to generate each dataset. The significance of experimental differences was evaluated by use of the Student’s *t* test (Prism 5.0). Results are expressed as mean ± SEM.

### Data sharing

RNA-seq data have been deposited to GEO with accession code GSE191005.

## Supplementary Information

**Additional file 1**. Supplementary figures, table, materials and methods.

## Data Availability

Not applicable.

## Abbreviations

MLL1: Mixed lineage leukemia 1
SEC: Super elongation complexes
MLL1-r: MLL1-rearranged
PROTAC: Proteolysis-targeting chimera
ALL: Acute lymphocytic leukemia
AML: Myeloid leukemia
CBX8: Chromobox homolog 8
BCoR: BCL-6 corepressor
ALPHA: Amplified luminescent proximity homogeneous assay
SDS-PAGE: Sodium dodecyl sulfate–polyacrylamide gel electrophoresis
D_max_: Maximal degradation
DC_50_: Concentration at which a protein is reduced by 50%
ChIP: Chromatin immunoprecipitation
GSEA: Gene set enrichment analysis
NES: Normalized enrichment score
