# Supplementary material for: A proteolysis-targeting chimera molecule selectively degrades ENL and inhibits malignant gene expression and tumor growth
Source: J Hematol Oncol. 2022 Apr 8;15:41. doi: 10.1186/s13045-022-01258-8 (PMC8994274; doi:10.1186/s13045-022-01258-8)
Supplement: Supplementary file 1 — Additional file 1. Supplementary figures, table, materials and methods. [file 13045_2022_1258_MOESM1_ESM.pdf]

Supplementary Materials

A Proteolysis-Targeting Chimera Molecule Selectively Degrades ENL and Inhibits Malignant Gene Expression and Tumor Growth

Xin Li,[1] Yuan Yao,[1] Fangrui Wu,[1] and Yongcheng Song [1, 2, *]

[1] Department of Pharmacology and Chemical Biology, [2] Dan L. Duncan Comprehensive Cancer Center, Baylor College of Medicine, 1 Baylor Plaza, Houston, TX 77030, USA.

Table of Contents:

Figure S1-S9	Page S2-S10
Table S1	Page S11
Compound synthesis	Page S12

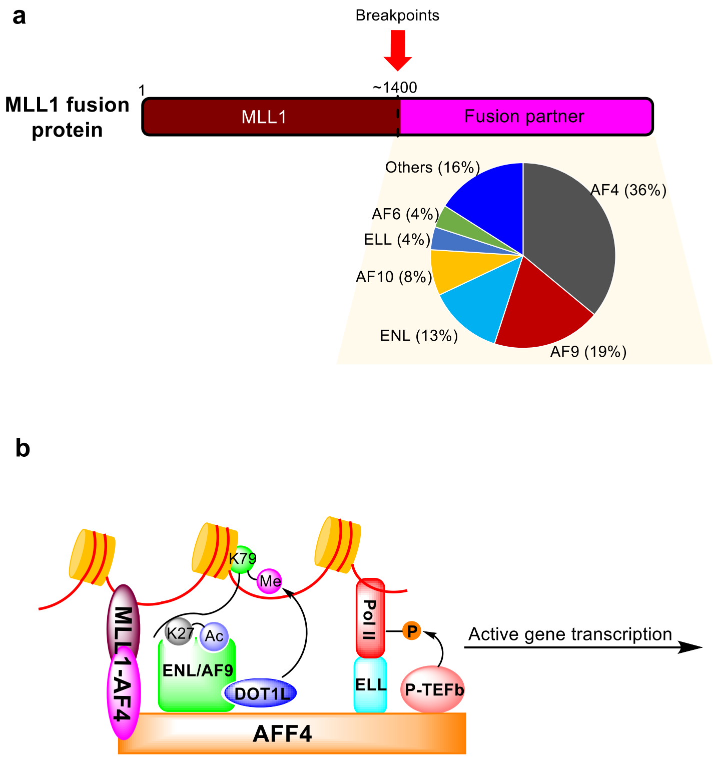

Figure S1. Illustration of MLL1 fusion oncoproteins and SEC. (**a**) MLL1 fusion proteins and frequencies of the MLL1-fusion partners in the clinic, with transcription cofactors AF4 (~36%), AF9 (~19%) and its paralog ENL (~13%), AF10 (~8%), ELL (~4%) and AF6 (~4%) being the most common (Ref. 12); (**b**) Schematic illustration of SEC recruited by MLL1-AF4 as well as its biological functions in gene transcription elongation. P-TEFb is the CDK9/cycline-T1 complex.

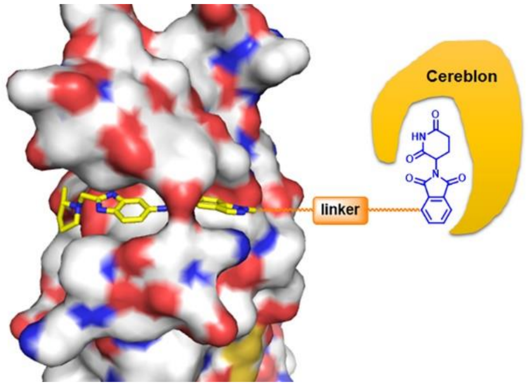

Figure S2. Structure-based design of ENL-targeting PROTAC molecules, which consist of a YEATS inhibitor SGC-iMLLT (tube model with C atoms in yellow which is bound to ENL; X-ray structure PDB: 6HT1) covalently linked with thalidomide (in blue), the ligand of E3 ubiquitin ligase Cereblon. ENL YEATS is shown as an electrostatic surface. The linking position is chosen for the linker to avoid steric conflicts with ENL.

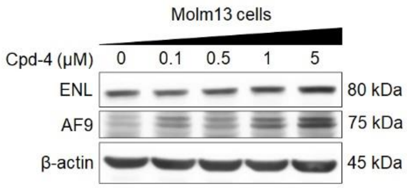

Figure S3. Levels of ENL, AF9 and β-actin (as a control) in Molm-13 cells upon treatment with compound **4** at the specified concentrations for 24h, showing it did not reduce these proteins levels.

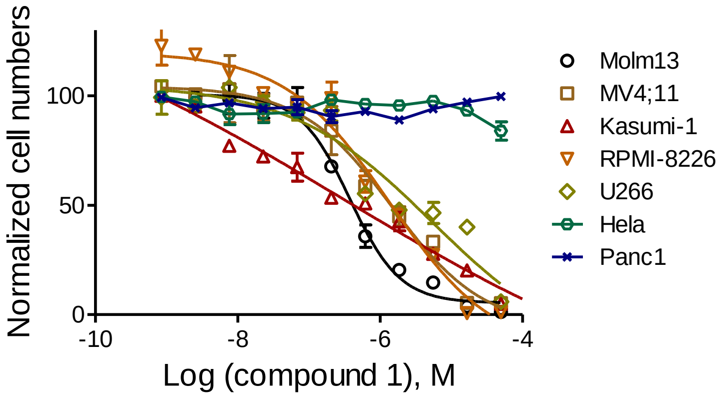

Figure S4. Dose-response curves for compound **1** to inhibit proliferation of cancer cells.

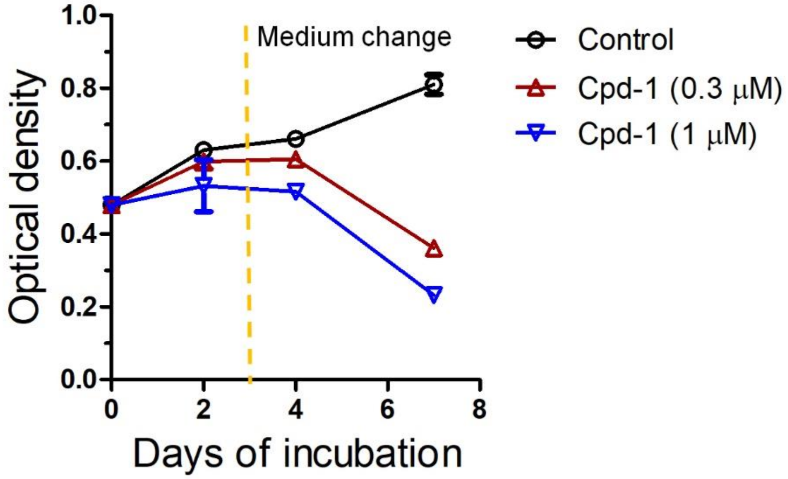

Figure S5. Raw growth curves of untreated (control) and Cpd-**1**-treated Molm-13 cells, using XTT assay.

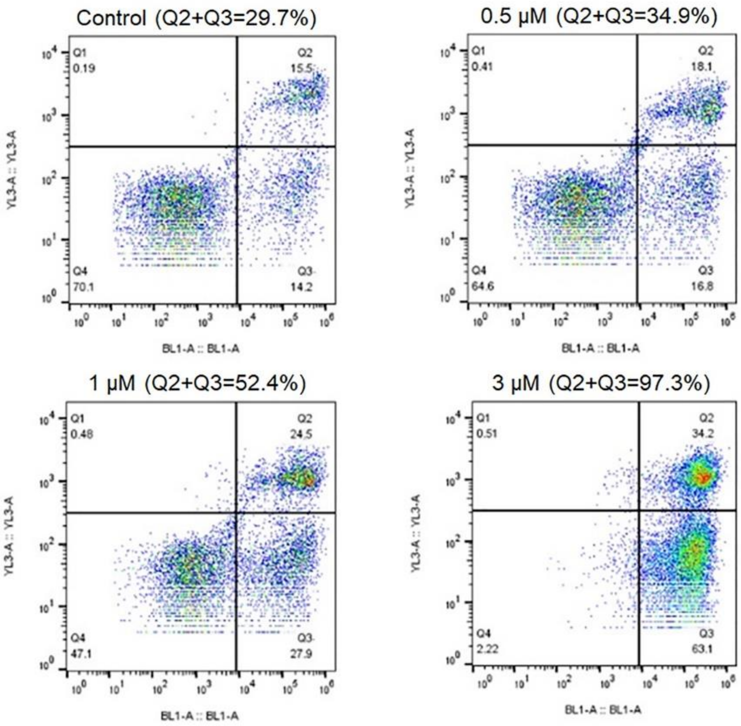

Figure S6. Compound **1** induced significant apoptosis of Molm-13 leukemia cells at 0.5, 1 and 3 μM (7-day incubation), as compared to the control. The upper right (Q2) number in each figure refers to the proportion of propidium iodide-positive, apoptosed cells and the lower right (Q3) number refers to that of annexin-V-positive cells (early apoptosis).

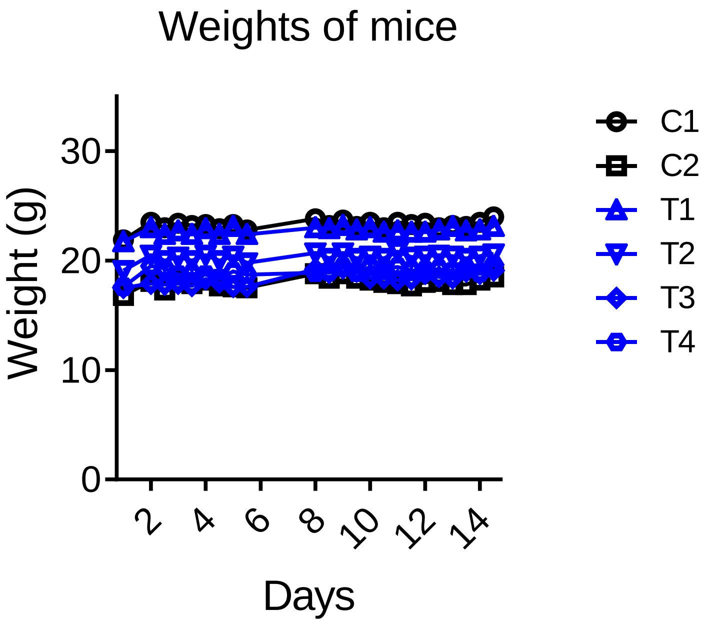

Figure S7. The weights of mice in the control (C1 and C2) and treatment groups (T1-4), showing compound **1** did not cause significant weight losses.

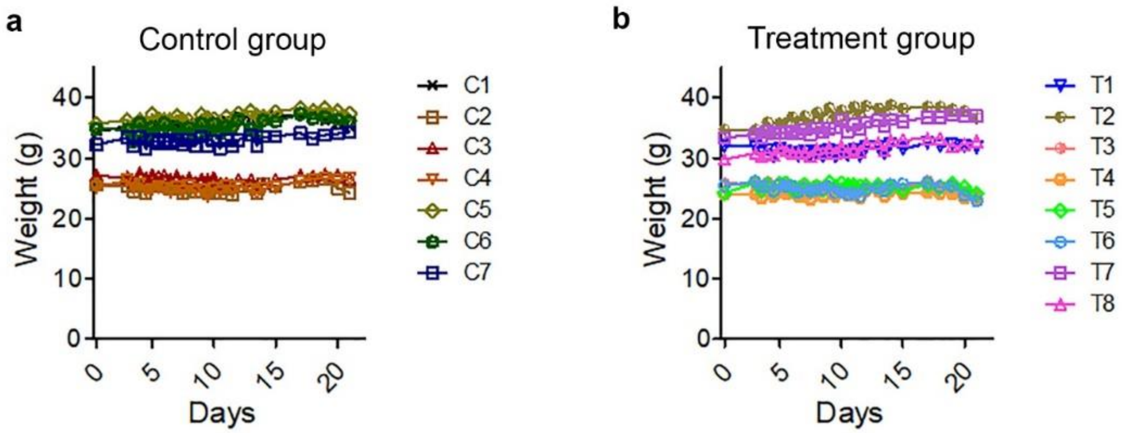

Figure S8. The weights of mice in the (**a**) control and (**b**) treatment groups, showing compound **1** did not cause significant weight losses.

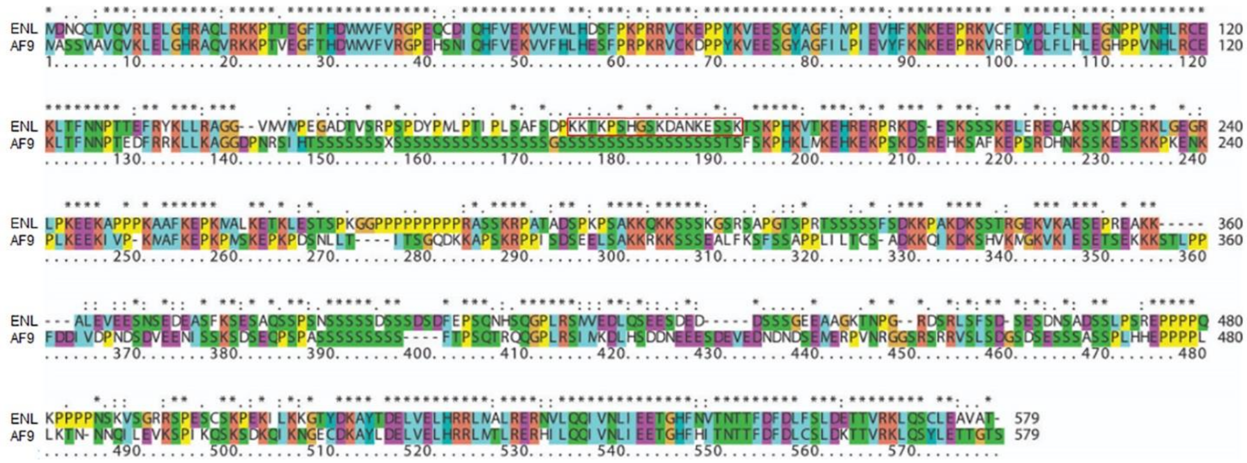

Figure S9. Protein sequence alignment of human ENL (upper) and AF9 (lower), using the program Clustal X2 (Larkin, M. A.; et al. Clustal W and Clustal X version 2.0. *Bioinformatics* **2007**, *23*, 2947-2948). While few different lysine residues are in the conserved YEATS (1-140) and AHD (499-) domains, ENL possesses 6 more lysine residues in a peptide segment 175-192 (red box) in the intrinsically disordered region.

Table S1. Biochemical inhibition (IC_{50}, nM) against the YEATS-H3K27ac interaction.

compound	ENL YEATS	AF9 YEATS
**1**	170 ± 74	750 ± 15
**2**	100 ± 43	317 ± 58
**3**	610 ± 190	58 ± 20
SGC-iMLLT	32 ± 1.2	11 ± 0.6

Compound synthesis

All chemicals for synthesis were purchased from Alfa Aesar (Ward Hill, MA), Aldrich (Milwaukee, WI) or Combi-Blcoks (San Diego, CA). The identity of the synthesized compounds was characterized by ^{1}H and ^{13}C NMR on a Varian (Palo Alto, CA) 400-MR spectrometer and mass spectrometer (Shimadzu LCMS-2020). The identity of SYC-2229 was confirmed with high resolution mass spectra (HRMS) using an Agilent 6550 iFunnel quadrupole-time-of-flight (Q-TOF) mass spectrometer with electrospray ionization (ESI). The purities of the final compounds were determined to be >95% with a Shimadzu Prominence HPLC using a Zorbax C18 (or C8) column (4.6 x 250 mm) monitored by UV at 254 nm. The synthesis of YEATS inhibitor SGC-iMLLT followed the previous report (Ref. 39). The synthetic methods for compounds **1-4** are shown in the following scheme.

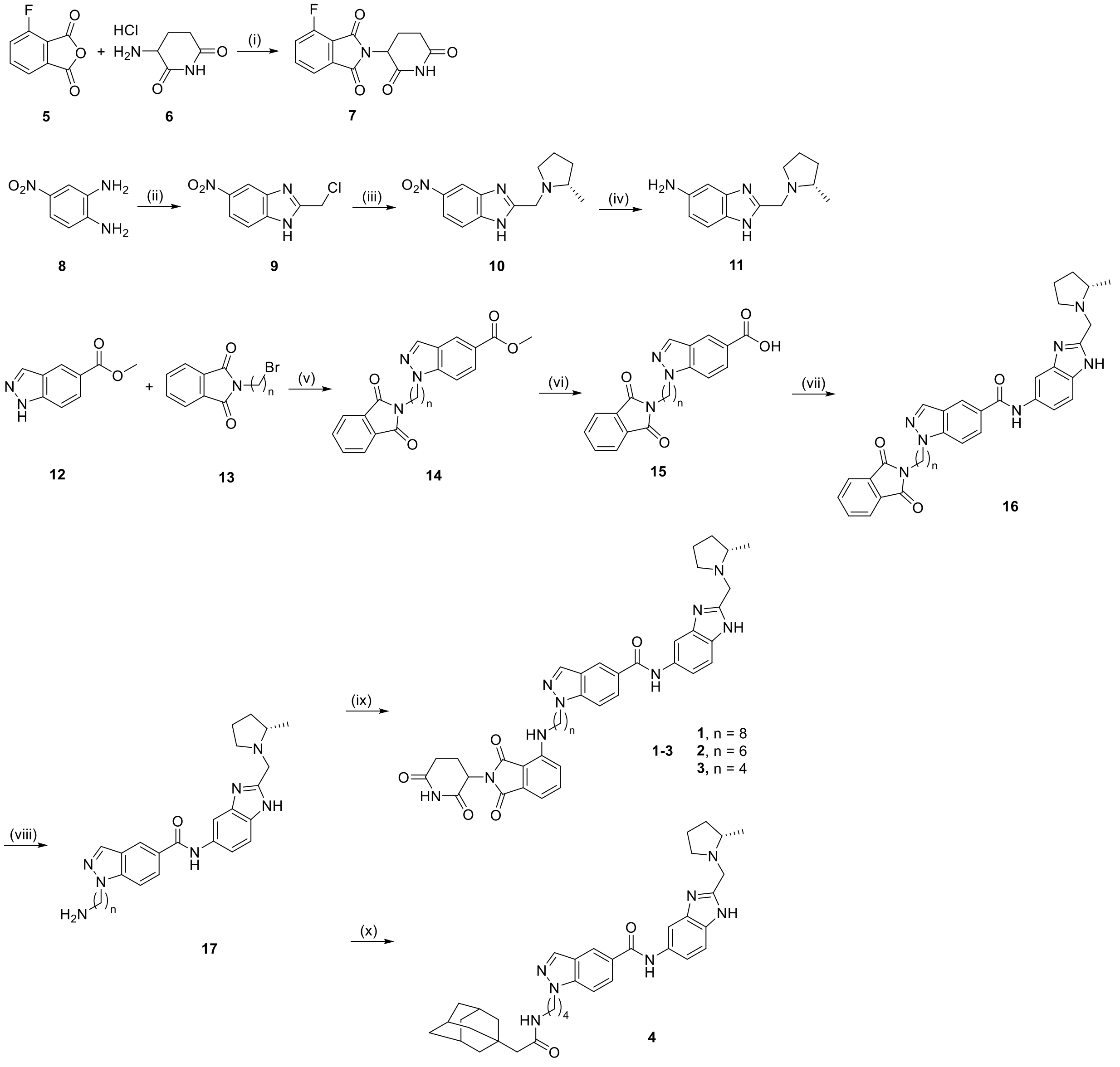

Reagents and conditions: (i) KOAc, AcOH, 120 °C, 12 h, 91.1%; (ii) Ethyl chloroacetate, 4N HCl solution, 100 °C, 48 h, 94.1%; (iii) (2*S*)-2-Methyl-pyrrolidine, Na_2CO_3, MeCN, 25 °C, 12 h then 60 °C, 3 h, 70.3%; (iv) 10% Pd-C, MeOH, H_2, 25 °C, 12 h, 100%; (v) NaH, DMF, 0 °C to 25 °C, 12 h, 17.3-47.7%; (vi) 37% HCl solution, 100 °C, 4 h, 78.4-100%; (vii) **11**, HATU, DIPEA, DCM, 25 °C, 12 h, 64.9-69.9%; (viii) NH_2NH_2, EtOH, 50 °C, 12 h, 79.7-95.9%; (ix) **7**, DIPEA, DMSO, 90 °C, 12 h, 40.3-43.6%; (x) 1-adamantaneacetic acid, HATU, DIPEA, DCM, 25 °C, 12 h, 64.4%.

A mixture of 3-fluorophthalic anhydride (**5**, 0.50 g, 3.01 mmol), 3-aminopiperidine-2,6-dione hydrochloride (**6**, 0.54 g, 3.31 mmol), and potassium acetate (0.89 g, 9.03 mmol) in acetic acid (10 mL) was heated to 120 °C for 12 h. Upon cooling to room temperature, the reaction was quenched with water. The mixture was extracted with ethyl acetate (3 x 20 mL) and the organic phase washed with brine (3 x 10 mL) and dried over Na_2SO_4. The solvent was removed under vacuum and the crude product purified with column chromatography (silica gel, hexanes/ethyl acetate, 2/1) to give compound **7** as an off-white solid (0.76 g, 91.1%). ^{1}H NMR (400 MHz, $CDCl_3$) δ 8.21 (s, 1H), 7.74 (dd, *J* = 24.3, 5.2 Hz, 2H), 7.43 (t, *J* = 7.9 Hz, 1H), 5.08 – 4.87 (m, 1H), 3.00 – 2.64 (m, 3H), 2.16 (d, *J* = 9.5 Hz, 1H).

To a solution of 4-nitrobenzene-1,2-diamine (**8**, 2.00 g, 13.06 mmol) in 4 N HCl (20 mL) was added ethyl 2-chloroacetate (2.40 g, 19.59 mmol) dropwise. The mixture was heated to 100 ºC for 48 h. The ammonium hydroxide was added dropwise at 0°C to give a precipitate, which was filtered, washed with cold water, and dried to give compound **9** as a brown solid (2.60 g), which was used without further purification. To a mixture of compound **9** (2.00 g, 9.45 mmol) and Na_2CO_3 (2.51 g, 23.63 mmol) in anhydrous CH_3CN (30 mL) was added (*S*)-2-methylpyrrolidine (0.97 g, 11.34 mmol). The reaction mixture was stirred at room temperature for overnight and then heated to 60 ^{0}C for 3 h. The residues were filtered, washed with acetone, concentrated, and purified with column chromatography (silica gel, dichloromethane/methanol, 30/1) to give compound **10** as a brown solid (1.73 g, 70.3%). ^{1}H NMR (400 MHz, $CDCl_3$) 8.46 (s, 1H), 8.15 – 8.05 (m, 1H), 7.57 (d, *J* = 8.8 Hz, 1H), 4.20 (d, *J* = 15.3 Hz, 1H), 3.71 (d, *J* = 15.3 Hz, 1H), 3.09 – 2.96 (m, 1H), 2.52 (dd, *J* = 13.8, 7.0 Hz, 1H), 2.30 (q, *J* = 8.8 Hz, 1H), 1.95 – 1.83 (m, 1H), 1.64 (dd, *J* = 14.5, 6.5 Hz, 2H), 1.31 (ddd, *J* = 16.9, 12.4, 8.6 Hz, 1H), 1.01 (d, *J* = 6.0 Hz, 3H). ^{13}C NMR (100 MHz, $CDCl_3$) δ 158.9, 143.3, 118.2, 60.2, 55.1, 51.7, 32.7, 21.8, 19.0.

A solution of compound **10** (1.25 g, 4.81 mmol) and Pd (10% on Carbon, 0.125 g) in MeOH (25 mL) was hydrogenated at room temperature for 12 h to produce compound **11** as a brown solid (1.12 g, 100%).

To the solution of methyl 1*H*-indazole-5-carbonate (**12**, 0.50 g, 2.84 mmol) in dry DMF (10 ml) was added NaH (60%, 0.17 g, 4.26 mmol) slowly at 0 ^{0}C and stirred for 30 min. A solution of compound **13** (3.69 mmol) in DMF (5 ml) was then added. The resulting mixture was stirred at room temperature for 12 h before quenching with a NH_4Cl solution and extracted with ethyl acetate (3 x 20 mL). The organic phase was washed with brine, dried over Na_2SO_4, concentrated, and purified with column chromatography (silica gel, hexanes/ethyl acetate, 5/1) to give compound **14** as a white solid in 17.3-47.7% yield. Compound **14** (2.36 mmol) in 37% hydrochloride acid (10 mL) was heated at 100 °C for 4 h. The reaction mixture was diluted with brine and extracted with ethyl acetate (3 x 20 mL). The organic phase was dried over Na_2SO_4 and concentrated to get compound **15** as a white solid.

For compound **15a**, ^{1}H NMR (400 MHz, $CDCl_3$) δ 8.58 (s, 1H), 8.12 (s, 1H), 8.08 (d, *J* = 8.7 Hz, 1H), 7.81 (dd, *J* = 5.0, 3.1 Hz, 2H), 7.67 (dd, *J* = 5.0, 3.1 Hz, 2H), 7.42 (d, *J* = 8.9 Hz, 1H), 4.38 (t, *J* = 6.8 Hz, 2H), 3.63 (t, *J* = 7.2 Hz, 2H), 1.91 (s, 2H), 1.62 (s, 2H), 1.29 (s, 8H). ^{13}C NMR (100 MHz, $CDCl_3$) δ 172.1, 168.5, 141.5, 134.8, 133.9, 132.2, 127.4, 125.8, 123.7, 123.2, 122.1, 109.0, 49.2, 38.0, 29.8, 29.1, 29.0, 28.6, 26.8.

For compound **15b**, ^{1}H NMR (400 MHz, $CDCl_3$) δ 8.59 (s, 1H), 8.12 (s, 1H), 8.08 (d, *J* = 8.9 Hz, 1H), 7.82 (dd, *J* = 5.0, 2.9 Hz, 2H), 7.73 – 7.66 (m, 2H), 7.42 (d, *J* = 8.9 Hz, 1H), 4.40 (t, *J* = 7.0 Hz, 2H), 3.65 (t, *J* = 7.1 Hz, 2H), 1.99 – 1.89 (m, 2H), 1.64 (dd, *J* = 13.8, 6.8 Hz, 2H), 1.41 – 1.32 (m, 4H). ^{13}C NMR (100 MHz, $CDCl_3$) δ 172.1, 168.6, 141.6, 134.9, 134.0, 132.2, 127.5, 125.9, 123.8, 123.3, 122.1, 109.0, 49.1, 37.9, 29.7, 28.5, 26.5.

For compound **15c**, ^{1}H NMR (400 MHz, $CDCl_3$) δ 8.59 (s, 1H), 8.12 (s, 1H), 8.09 (dd, *J* = 8.9, 1.3 Hz, 1H), 7.83 (dd, *J* = 5.4, 3.0 Hz, 2H), 7.71 (dd, *J* = 5.4, 3.0 Hz, 2H), 7.47 (d, *J* = 8.9 Hz, 1H), 4.47 (t, *J* = 6.9 Hz, 2H), 3.73 (t, *J* = 6.9 Hz, 2H), 2.06 – 1.94 (m, 2H), 1.77 – 1.65 (m, 2H). ^{13}C NMR (100 MHz, $CDCl_3$) δ 171.0, 168.6, 141.7, 135.1, 134.2, 132.2, 127.6, 125.9, 123.8, 123.4, 121.9, 109.0, 48.5, 37.3, 27.1, 26.0.

A mixture of compound **11** (0.68 g, 2.94 mmol), compound **15** (2.45 mmol), diisopropylethylamine (0.63 g, 4.90 mmol) and HATU (1-[Bis(dimethylamino)methylene]-1H-1,2,3-triazolo[4,5-b]pyridinium 3-oxide hexafluorophosphate, 1.12 g, 2.94 mmol) in dichloromethane (25 mL) was stirred vigorously for 12 h. It was diluted with dichloromethane (25 mL), washed with brine, dried over Na_2SO_4, concentrated, and purified with column chromatography (silica gel, dichloromethane/methanol, 20/1) to give compound **16** as a pale yellow solid in 64.9-69.9% yield.

For compound **16a**, ^{1}H NMR (400 MHz, $CDCl_3$) δ 8.77 (s, 1H), 8.30 (s, 1H), 8.08 (s, 1H), 7.96 (s, 1H), 7.91 (d, *J* = 8.7 Hz, 1H), 7.82 – 7.71 (m, 2H), 7.66 (dd, *J* = 5.0, 3.1 Hz, 2H), 7.48 (d, *J* = 8.3 Hz, 1H), 7.35 (dd, *J* = 18.2, 8.3 Hz, 2H), 4.31 (d, *J* = 6.5 Hz, 2H), 4.10 (d, *J* = 14.7 Hz, 1H), 3.70 – 3.48 (m, 3H), 2.97 (d, *J* = 4.6 Hz, 1H), 2.55 (d, *J* = 6.0 Hz, 1H), 2.35 – 2.23 (m, 1H), 1.98 – 1.75 (m, 3H), 1.62 (dd, *J* = 39.2, 6.2 Hz, 4H), 1.39 (d, *J* = 6.1 Hz, 1H), 1.23 (s, 8H), 1.06 (d, *J* = 5.7 Hz, 3H).

For compound **16b**, ^{1}H NMR (400 MHz, $CDCl_3$) δ 8.45 (s, 1H), 8.29 (s, 1H), 8.11 (s, 1H), 8.00 (s, 1H), 7.90 (d, *J* = 8.8 Hz, 1H), 7.80 (dd, *J* = 5.1, 3.0 Hz, 2H), 7.68 (dd, *J* = 5.1, 3.0 Hz, 2H), 7.53 (d, *J* = 8.5 Hz, 1H), 7.40 (d, *J* = 8.8 Hz, 1H), 7.30 (d, *J* = 7.7 Hz, 1H), 4.36 (t, *J* = 6.9 Hz, 2H), 4.12 (d, *J* = 14.9 Hz, 1H), 3.63 (dd, *J* = 16.9, 10.4 Hz, 3H), 3.00 (t, *J* = 7.3 Hz, 1H), 2.56 (d, *J* = 6.8 Hz, 1H), 2.31 (q, *J* = 8.8 Hz, 1H), 1.92 (dd, *J* = 18.5, 11.9 Hz, 3H), 1.71 (dd, *J* = 11.1, 3.9 Hz, 2H), 1.65 – 1.53 (m, 2H), 1.45 (d, *J* = 6.2 Hz, 1H), 1.31 (s, 4H), 1.09 (d, *J* = 6.0 Hz, 3H). ^{13}C NMR (100 MHz, $CDCl_3$) δ 168.6, 166.4, 154.2, 140.7, 134.2, 134.0, 133.4, 132.2, 127.9, 125.4, 123.7, 123.3, 121.3, 116.2, 109.3, 60.5, 55.1, 51.9, 49.1, 37.9, 32.9, 29.7, 28.5, 26.4, 21.9, 19.1.

For compound **16c**, ^{1}H NMR (400 MHz, $CDCl_3$) δ 8.41 (s, 1H), 8.27 (s, 1H), 8.10 (s, 1H), 7.99 (s, 1H), 7.89 (d, *J* = 8.4 Hz, 1H), 7.79 (s, 2H), 7.68 (d, *J* = 1.9 Hz, 2H), 7.53 (d, *J* = 8.3 Hz, 1H), 7.43 (d, *J* = 8.6 Hz, 1H), 7.29 (s, 1H), 4.43 (t, *J* = 6.5 Hz, 2H), 4.12 (d, *J* = 15.0 Hz, 1H), 3.68 (dd, *J* = 19.6, 11.7 Hz, 3H), 3.01 (t, *J* = 7.0 Hz, 1H), 2.58 (dd, *J* = 19.6, 13.0 Hz, 1H), 2.32 (dd, *J* = 17.3, 8.5 Hz, 1H), 2.01 – 1.88 (m, 3H), 1.77 – 1.61 (m, 4H), 1.44 (t, *J* = 18.5 Hz, 1H), 1.10 (d, *J* = 5.9 Hz, 3H). ^{13}C NMR (100 MHz, $CDCl_3$) δ 168.5, 166.3, 154.2, 140.7, 134.5, 134.1,

133.4, 132.1, 128.0, 125.5, 123.7, 123.4, 121.2, 116.2, 109.3, 60.5, 55.2, 51.9, 48.4, 37.3, 32.9, 29.8, 27.1, 25.9, 22.0, 19.2.

A mixture of compound **16** (0.55 mmol) and anhydrous hydrazine (0.07 g, 2.22 mmol) in EtOH (10 mL) was heated to 50 °C for 12 h. It was diluted with dichloromethane (50 mL), washed with brine, dried over Na_2SO_4, and concentrated to get compound **17**, which was used without further purification. A mixture of compound **17 (**0.40 mmol), compound **7** (0.13 g, 0.48 mmol) and diisopropylethylamine (0.10 g, 0.80 mmol) in DMSO (5 mL) was heated to 90 ^{0}C for 12 h. it was then diluted with dichloromethane (20 mL) and water (20 mL) and extracted with dichloromethane (3 x 20 mL). The organic phase was washed with brine, dried over Na_2SO_4, concentrated, and purified with column chromatography (silica gel, dichloromethane/methanol, 20/1) to give compounds **1-3** as a pale yellow solid in 40.3-43.6% yield.

***N*-{2-[[(2S)-2-methyl-1-pyrrolidinyl]methyl]-1H-benzimidazole-5-yl)-1-(8-(2-(2,6-dioxo-3-piperidinyl)-4-fluoro-1*H*-Isoindole-1,3(2*H*)-dione-4-yl)-octyl)-1*H*-indazole-5-carboxamide (1).** ^{1}H NMR (400 MHz, $CDCl_3$) δ 8.39 (s, 1H), 8.30 (s, 1H), 8.06 (d, *J* = 11.6 Hz, 2H), 7.93 (d, *J* = 8.8 Hz, 1H), 7.54 (d, *J* = 8.4 Hz, 1H), 7.43 (dd, *J* = 8.1, 4.5 Hz, 2H), 7.35 (s, 1H), 7.04 (d, *J* = 7.1 Hz, 1H), 6.83 (d, *J* = 8.6 Hz, 1H), 6.25 – 6.14 (m, 1H), 5.29 (s, 1H), 4.90 (dd, *J* = 12.0, 5.3 Hz, 1H), 4.38 (t, *J* = 6.9 Hz, 2H), 4.22 (dd, *J* = 14.6, 2.9 Hz, 1H), 3.86 – 3.74 (m, 1H), 3.19 (d, *J* = 5.8 Hz, 2H), 3.09 (t, *J* = 8.4 Hz, 1H), 2.87 – 2.66 (m, 4H), 2.48 (dd, *J* = 17.7, 8.7 Hz, 1H), 2.07 (ddd, *J* = 12.6, 7.5, 4.2 Hz, 2H), 1.96 – 1.87 (m, 1H), 1.80 (ddd, *J* = 18.6, 12.5, 7.1 Hz, 2H), 1.62 – 1.48 (m, 3H), 1.30 – 1.21 (m, 9H), 1.16 (d, *J* = 6.1 Hz, 3H). ^{13}C NMR (100 MHz, $CDCl_3$) δ 171.4, 169.7, 169.0, 168.9, 167.8, 166.2, 153.8, 147.1, 140.8, 136.2, 134.3, 133.4, 132.6, 128.0, 125.4, 123.7, 121.2, 116.8, 111.5, 109.9, 109.4, 60.8, 55.1, 55.0, 52.0, 49.2, 49.1, 42.7, 32.9, 31.6, 29.8, 29.7, 29.2, 29.1, 29.0, 26.8, 26.6, 23.0, 22.0, 19.0. HRMS (ESI) m/z: $[M + H]^+$ calcd for $C_{42}H_{47}N_9O_5$ 758.37729; found 758.37799.

***N*-{2-[[(2S)-2-methyl-1-pyrrolidinyl]methyl]-1H-benzimidazole-5-yl)-1-(6-(2-(2,6-dioxo-3-piperidinyl)-4-fluoro-1*H*-Isoindole-1,3(2*H*)-dione-4-yl)-hexyl)-1*H*-indazole-5-carboxamide (2).** ^{1}H NMR (400 MHz, $CDCl_3$) δ 8.31 (s, 1H), 8.17 (s, 1H), 8.08 (s, 1H), 7.92 (d, *J* = 8.6 Hz, 1H), 7.67 – 7.55 (m, 1H), 7.49 – 7.41 (m, 3H), 7.07

(d, *J* = 7.0 Hz, 1H), 6.82 (d, *J* = 8.5 Hz, 1H), 6.17 (s, 1H), 4.91 (dd, *J* = 11.9, 5.0 Hz, 1H), 4.42 (t, *J* = 6.5 Hz, 2H), 4.15 (d, *J* = 15.0 Hz, 1H), 3.73 – 3.58 (m, 1H), 3.24 – 3.10 (m, 2H), 3.02 (dd, *J* = 11.1, 5.9 Hz, 1H), 2.88 – 2.65 (m, 2H), 2.55 (ddd, *J* = 18.7, 11.8, 4.7 Hz, 1H), 2.34 (dd, *J* = 17.0, 8.1 Hz, 1H), 2.10 (dd, *J* = 11.1, 4.0 Hz, 1H), 2.03 – 1.88 (m, 4H), 1.82 – 1.70 (m, 2H), 1.64 – 1.54 (m, 2H), 1.42-1.40 (m, 1H), 1.25 (s, 4H), 1.13 (d, *J* = 6.0 Hz, 3H). ^{13}C NMR (100 MHz, $CDCl_3$) δ 171.5, 169.7, 168.9, 167.8, 166.2, 147.1, 140.8, 136.3, 134.4, 134.1, 132.6, 130.0, 127.9, 125.4, 123.8, 121.5, 116.8, 111.6, 110.0, 109.4, 56.1, 53.7, 51.0, 49.1, 49.0, 42.5, 32.2, 32.1, 31.6, 29.7, 29.5, 29.2, 26.5, 22.9, 22.8, 21.7, 19.3. MS (ESI) m/z: $[M + H]^+$ calcd for $C_{40}H_{44}N_9O_5$ 730.34; found 730.3.

***N*-{2-[[(2S)-2-methyl-1-pyrrolidinyl]methyl]-1H-benzimidazole-5-yl)-1-(4-(2-(2,6-dioxo-3-piperidinyl)-4-fluoro-1*H*-Isoindole-1,3(2*H*)-dione-4-yl)-butyl)-1*H*-indazole-5-carboxamide (3).** ^{1}H NMR (400 MHz, $CDCl_3$) δ 8.47 (s, 1H), 8.28 (s, 1H), 8.09 (s, 1H), 8.02 (s, 1H), 7.89 (d, *J* = 8.2 Hz, 1H), 7.54 (d, *J* = 6.7 Hz, 1H), 7.43 – 7.30 (m, 3H), 7.02 (d, *J* = 7.0 Hz, 1H), 6.73 (d, *J* = 8.5 Hz, 1H), 6.15 (d, *J* = 5.8 Hz, 1H), 4.89 (dd, *J* = 11.5, 4.9 Hz, 1H), 4.42 (s, 2H), 4.19 (d, *J* = 14.7 Hz, 1H), 3.74 (d, *J* = 14.7 Hz, 1H), 3.20 (d, *J* = 5.9 Hz, 2H), 3.04 (d, *J* = 6.9 Hz, 1H), 2.87 – 2.59 (m, 4H), 2.14 – 2.02 (m, 5H), 1.85 – 1.45 (m, 5H), 1.13 (d, *J* = 5.9 Hz, 3H). ^{13}C NMR (100 MHz, $CDCl_3$) δ 171.5, 169.6, 169.2, 169.1, 167.7, 166.2, 153.2, 146.7, 140.6, 136.2, 134.5, 133.5, 132.5, 130.0, 128.1, 125.6, 123.8, 121.5, 116.7, 111.7, 110.1, 109.1, 61.0, 56.1, 54.9, 51.8, 49.1, 48.8, 42.0, 32.8, 31.6, 29.4, 26.9, 26.3, 22.9, 21.9, 18.8. MS (ESI) m/z: $[M + H]^+$ calcd for $C_{38}H_{40}N_9O_5$ 702.31; found 702.3.

***N*-{2-[[(2S)-2-methyl-1-pyrrolidinyl]methyl]-1H-benzimidazole-5-yl)-1-(4-(2-(adamantaneacetyl-1-carboxamido)-butyl)-1*H*-indazole-1-carboxamide (4).** A mixture of compound **17c** (0.03 g, 0.07 mmol), 1-adamantaneacetic acid (0.02 g, 0.08 mmol), diisopropylethylamine (0.02 g, 0.14 mmol and HATU (0.03 g, 0.08 mmol) in dichloromethane (1 mL) was stirred for 12 h. It was diluted with dichloromethane (10 mL), washed with brine, dried over Na_2SO_4, concentrated, and purified with column chromatography (silica gel, dichloromethane/methanol, 20/1) to give compound **4** as a white solid in 64.4% yield. ^{1}H NMR (400 MHz, $CDCl_3$) δ 8.74 (s, 1H), 8.30 (s, 1H), 8.12 (s, 1H), 7.99 (s, 1H), 7.91 (d, *J* = 8.8 Hz, 1H), 7.53 (d, *J* = 7.8 Hz, 1H), 7.37 (d, *J* = 8.8 Hz, 2H), 5.58 (t, *J* = 5.5 Hz, 1H), 4.34 (t, *J* = 6.8 Hz, 2H), 4.13 (d, *J* = 14.9 Hz, 1H), 3.67 (d, *J* = 14.9 Hz, 1H), 3.13 (dd, *J* = 13.2, 6.7 Hz, 2H), 3.06 – 2.98 (m, 1H), 2.60 – 2.50 (m, 1H), 2.34 (q, *J* = 8.8 Hz, 1H), 2.02 – 1.83

(m, 8H), 1.88 – 1.62 (m, 3H), 1.62 – 1.50 (m, 9H), 1.46 – 1.36 (m, 3H), 1.30 – 1.25 (m, 2H), 1.10 (d, *J* = 6.0 Hz, 3H). ^{13}C NMR (100 MHz, $CDCl_3$) δ 171.1, 166.5, 153.8, 140.7, 134.2, 133.6, 128.1, 125.6, 123.7, 121.4, 116.4, 109.2, 60.5, 55.0, 54.0, 52.0, 51.8, 49.0, 42.8, 39.2, 36.9, 32.9, 32.8, 31.9, 29.8, 29.7, 29.6, 29.4, 28.7, 26.4, 21.9, 19.1. MS (ESI) m/z: $[M + H]^+$ calcd for $C_{37}H_{48}N_7O_2$ 622.38; found 622.4.
